# The expression and biological effect of NR2F6 in non-small cell lung cancer

**DOI:** 10.3389/fonc.2022.940234

**Published:** 2022-09-02

**Authors:** Shu lin Yang, Huan qin Guan, Hong bao Yang, Yao Chen, Xiao ying Huang, Lei Chen, Zhi fa Shen, Liang xing Wang

**Affiliations:** ^1^ Key Laboratory of Laboratory Medicine, Ministry of Education of China, Zhejiang Provincial Key Laboratory of Medicine Genetics, School of Laboratory Medicine and Life Sciences, Wenzhou Medical University, Wenzhou, China; ^2^ Department of pathology, The First Affiliated Hospital of Wenzhou Medical University, Wenzhou, China; ^3^ Department of Pulmonary and Critical Care Medicine, The First Affiliated Hospital of Wenzhou Medical University, Wenzhou, China

**Keywords:** None small lung cancer, the cancer genome atla, nuclear receptor subfamily 2 group f member 6, heterogeneous nuclear ribonucleoprotein D, prognosis

## Abstract

**Objective:**

This study aimed to explore the expression and effect of the nuclear receptor subfamily 2 group F member 6 (NR2F6) gene in non-small cell lung cancer (NSCLC) and provide an experimental basis for the targeted therapy of NSCLC.

**Method:**

First, the expression of NR2F6 in lung cancer tissues was analyzed using the Gene Expression Omnibus and the Cancer Genome Atlas (TCGA) databases, and the expression of NR2F6 in lung cancer tissues and cells was verified by Western blotting and quantitative polymerase chain reaction. Next, the relationship between NR2F6 expression and the clinicopathological features of lung cancer was analyzed *via* immunohistochemistry, and the relationship between NR2F6 expression and prognosis was analyzed using the Kaplan–Meier Plotter. The influence of NR2F6 knockdown on the proliferation capacity of lung cancer cells was then verified at cell level. Finally, the expression of heterogeneous nuclear ribonucleoprotein D (HNRNPD) in lung cancer tissue was analyzed using the TCGA database and immunohistochemistry. The impact of HNRNPD knockdown on the proliferation capacity of lung cancer cells was verified at cell level, and the relationship between NR2F6 and HNRNPD was verified by co-immunoprecipitation.

**Results:**

NR2F6 was highly expressed in lung cancer tissues and cells, and its expression was positively correlated with the depth of invasion, lymphatic metastasis, and clinical stage of lung cancer. High expression of NR2F6 in lung cancer was also significantly associated with poor prognosis. At cell level, NR2F6 knockdown was found to inhibit the proliferation of H460 and H358 in lung cancer cells. Furthermore, the TCGA database and immunohistochemical results showed that HNRNPD was highly expressed in lung cancer tissues and was highly consistent with NR2F6 expression in these tissues. Knockdown of HNRNPD also inhibited the proliferation of lung cancer cells. The co-immunoprecipitation experiment verified that NR2F6 interacted with HNRNPD.

**Conclusion:**

NR2F6 may interact with HNRNPD to jointly regulate the progression of lung cancer, and this conclusion provides a new experimental basis for the study of the molecular targeted therapy of NSCLC.

## Introduction

Lung cancer is the most common malignant tumor in the world and has the highest mortality rate (1). It can be divided into non-small cell lung cancer (NSCLC) and small cell lung cancer (SCLC) according to different pathological types, with NSCLC accounting for 85% of all types of lung cancer (2, 3). NSCLC mainly includes lung adenocarcinoma(LUAD) and lung squamous cell arcinoma(LUSC).There are often no obvious symptoms in the early stage of lung cancer (4), and most confirmed cases are already in the advanced stage (2). Despite the progress and improvement of surgery and drug therapy, the five-year survival rate for patients with lung cancer remains low (5). Thus, there is an urgent need to explore the molecular mechanisms of NSCLC progression and seek new therapeutic targets with moderate response to improve the survival rate of patients.

In 2018, Klepsch et al. (6) reported a transcription factor nuclear receptor subfamily 2 group F member 6 (NR2F6) in nature, arousing great interest in the field. NR2F6 is a member of the chicken ovalbumin upstream promoter-transcription factors family. It was initially identified in human fetal liver and mouse embryos (7, 8). NR2F6 has a nuclear receptor DNA-binding domain and ligand-binding domain, but an endogenous ligand has not yet been found. Thus, this nuclear receptor is an orphan nuclear receptor and plays an important role in various diseases and cellular homeostasis, including cancer (9–14).

The expression of NR2F6 is significantly up-regulated in a variety of malignant tumors, such as acute myeloid leukemia (AML) (15), breast cancer (16), Head and neck squamous cell carcinoma (17), colon cancer (18), and lymphoma (19). Ichim et al. (15) found that, in AML cells with growth potential, the messenger RNA (mRNA) transcription level of NR2F6 was four times higher than that of cells without growth potential (13). NR2F6 expression level was also found to be higher in long-acting hematopoietic stem cells than in short-acting hematopoietic stem cells and progenitor cells, and its expression level was low in induced differentiated AML cells. Furthermore, Li et al. (20) found that the expression of NR2F6 was significantly up-regulated in ovarian cancer and that the high expression of NR2F6 was closely related to cisplatin resistance in ovarian cancer cells.

Currently, there are no reports on whether NR2F6 is related to the progression of lung cancer, although previous studies have shown that (6, 18, 21) it may play a role in actively regulating the survival rate of tumor cells and inducing cancer progression. After establishing mouse subcutaneous B16-OVA and MC38 colon cancer tumor models, Klepsch et al. (6) found that the knockout of NR2F6 enhanced the activity of an established programmed death-ligand 1 (PD-L1) checkpoint blockade, thereby promoting tumor regression, and increasing the survival rate of the subcutaneous tumor model. Using a transgenic adenocarcinoma mouse prostate (TRAMP) cancer model, Natascha et al. (21) found that NR2F6 gene knockout could significantly improve patient survival rate. Furthermore, when NR2F6 was knocked out alone or together with PD-L1, maladjusted target genes in T lymphocytes in tumors showed favorable transcriptional changes, and Li et al. (18) found that, when NR2F6 was knocked down, the subcutaneous tumorigenicity of colon cancer cells in nude mice was significantly lower than that in the control group. However, Yang et al. (22) found that NR2F6 was highly expressed in normal human breast tissues, which inhibited the expression of aromatase protein (converting androgens into estrogen and stimulating the growth of breast cancer cells), and Klepsch et al. (6) found that NR2F6 acted as a safeguard to protect intestinal barrier homeostasis and played an important role in maintaining stem cell phenotypes (4).These studies suggest that NR2F6 may be closely related to the pathophysiology of malignant diseases and may play a dual role in cancer progression.

Overall, NR2F6 has been proven to be closely associated with the progression of multiple malignant tumors, such as cervical cancer, colon cancer, and breast cancer. However, the effect of NR2F6 on the progression of NSCLC is still unclear. The present study aimed to investigate the expression and molecular mechanism of NR2F6 in NSCLC and provide an experimental basis for the targeted therapy of NSCLC.

## Materials and methods

### Analysis using the Gene Expression Omnibus and the Cancer Genome Atlas data sets

The mRNA expression of NR2F6 in the NSCLC GSE17558, GSE32863, and GSE75037 data sets was downloaded from the GEO database (www.ncbi.nlm.nih.gov/geo). These three mRNA microarray data sets contained the mRNA microarray data of 147 lung cancer tumor samples and 147 paired para-carcinoma tissue samples. The standardized NR2F6 mRNA data and clinicopathological data of lung cancer in TCGA were downloaded from the UCSC Xena database (http://xena.ucsc.edu). A total of 59 lung adenocarcinoma tissue samples, 526 para-cancer tissue samples, 49 squamous cell lung carcinoma tissue samples, and 501 para-carcinoma tissue samples were included in this study.

### Survival analysis

The Kaplan–Meier Plotter (http://kmplot.com/analysis/index.php) and TCGA datebets were used to analyze the relationship between NR2F6 expression and prognosis. The tumor type was set as LUAD and LUSC, and the gene symbol was set as NR2F6. A Kaplan–Meier plot was drawn, and the prognostic analysis chart of NR2F6 in LUAD and LUSC was generated.Meanwhile,the prognostic analysis chart of NR2F6 in LUAD and LUSC was drawed. Furthermore,the NR2F6 expression and prognosis on TCGA date sets was drawed.

### Patient information and tissue specimens

Tissue samples were collected from 50 patients with NSCLC at the First Affiliated Hospital of Wenzhou Medical University between January 2018 and June 2019, and the paired normal para-carcinoma tissues were obtained. The specimens were immediately placed in cryopreservation tubes, frozen in liquid nitrogen, and stored at –80°C. At the same time, the clinical data of the patients were collected. Paraffin tissue samples were also collected from 200 patients with lung cancer who underwent surgical treatment in the hospital in 2018 and 2019; these samples were made into 12 tissue chips for immunohistochemical detection.

The study was approved by the Ethics Committee of the First Affiliated Hospital of Wenzhou Medical University, and all patients enrolled in the study signed the informed consent form. The use and ethics of lung cancer tissue specimens were approved by the committee, with the reference number YS2018-106.

### Cell line and cell culture

Six lung cancer cell lines (A549, H1650, PC-9, H460, H1975, and H358) and one normal bronchial epithelial cell line (Beas-2B) were purchased from the cell bank of the Chinese Academy of Sciences (Shanghai, China). All cell lines were cultured in RMI 1640 basal medium, to which 10% fetal bovine serum and 1% antibiotics (100 μg/mL streptomycin and 100 units/mL penicillin) were added. All cells were cultured in a CO_2_ cell incubator at 37°C and at a volume fraction of 5%. Routine tests were performed to confirm that the cells were not infected with mycoplasma.

### Plasmid, lentivirus construction, and cell transfection (H460)

In the logarithmic phase, the H460 cell line was inoculated into a six-well plate, and 1640 complete culture medium containing double antibodies was used to culture the cell line in a CO_2_ cell incubator at 37°C, 5% volume fraction. On the second day, fresh 1640 complete culture medium was replaced when the cell fusion in the six-well plate reached 50%. The virus containing the target NR2F6 gene (NR2F6#1, #2, and 3# sequences) and the negative control virus were added respectively to the medium to achieve the final concentration of the virus (6 µg/mL). The cells were further cultured in the incubator. The complete medium was replaced 12 hours after transfection, and the culturing of the cells continued in the CO_2_ incubator at 37°C and 5% volume fraction. The passage was carried out when cell fusion reached 80–90%. Cell strains were screened with 2 μg/mL puromycin, with stably transfected cell strains being screened in 7–10 days. The expression levels of NR2F6 protein and mRNA were determined using Western blotting and quantitative polymerase chain reaction (qPCR). The cell lines successfully down-regulated by the NR2F6 gene were represented as the shNR2F6#1, shNR2F6#2, and shNR2F6#3 groups, and the control cells were represented as the shNC group.

### Cell proliferation test

The stably transfected H460 cells were inoculated into a 96-well culture plate at a density of 2 × 10^6^/mL, before being cultured in a CO_2_ incubator at 37°C and 5% volume fraction. This was repeated in five wells of cells in both the experimental group and the control group, and 10 μL cholecystokinin octapeptide (CCK-8) reagent was added into each well every 24 hours. After the cells were cultured for 0 hours, 24 hours, 48 hours, and 72 hours, respectively, 10 μL CCK-8 solution was added into each well in the 96-well culture plate. The cells were then placed in a 37°C incubator for 2 hours. The absorbance of the cell samples was determined at 450 nm. The determination was repeated three times.

### Colony formation test

A total of 800 stably transfected H460 and H358 cells were cultured in 5 mL RPMI 1640 medium for 7–10 days until macroscopic clones were observed in a 6-cm culture dish. The medium was discarded, and the cells were washed twice with phosphate buffer solution (PBS). The cells were then fixed with 4% paraformaldehyde for 20 minutes at room temperature and stained with 0.1% crystal violet for 15 minutes at room temperature. An inverted microscope was then used to observe and count bacterial colonies. Image J (version 1.51) software was used for analysis.

### Western blot analysis

All cells and fresh tissues were washed three times with cold PBS and lysed in radioimmunoprecipitation assay buffer. They were then lysed with a mixture of protease inhibitors at 4°C for 10 minutes. A bicinchoninic acid assay kit was used to obtain the concentration of the denatured protein, after which protein of equal concentration (20 μg per well) was added, and the samples were separated by 10% sodium dodecyl sulphate–polyacrylamide gel electrophoresis gel. Next, the samples were transferred to a polyvinylidene fluoride membrane, which was sealed with 5% skim milk for 2 hours at room temperature and then incubated overnight at 4°C with NR2F6 (1:1000, Abcam, ab223265) and tubulin (#2148) primary antibodies. The strips were then incubated at room temperature for 2 hours in the corresponding secondary antibody (1:1000 A0208) coupled with Horseradish Peroxidase. The strips were cleaned with Tris-buffered saline solution (TBST) three times. After 10 minutes, they were observed using an enhanced chemiluminescence system. The expression of related proteins was quantified and analyzed using Image J software. Glyceraldehyde 3-phosphate dehydrogenase (1:1000, Cell Signaling Technology, #5174) and tubulin (#2148) were used for internal reference.

### qPCR analysis

Tissues and cells were collected to extract the total RNA according to the instructions included in the Trizol kit. The concentration and purity of total RNA was measured and retro-transcribed into cDNA using 1 μg total RNA and 20 μL total volume. The instructions for the qPCR reagent were followed, with the reaction system of 20 μL, and three parallel wells were used for each sample.

PCR reaction process: denaturation at 95°C for 5 minutes, then denaturation at 95°C for 15 seconds, annealing at 60°C for 30 seconds, and 72°C primer extension for 35 cycles. Using β-actin as an internal reference, data processing was undertaken using the relative transcript level of 2-ΔΔCt. The primer sequences were as follows: NR2F6 forward AGTCGAGCGGCAAGCAAGCATTAC, NR2F6 reverse GATCTGGCAGTCACGGTTGG, β-actin forward ATAGCACAGCCTGGATAGCAACGTAC, β-actin reverse CACCTTCTACAATGAGCTGCGTGTG.

### Tissue microarray preparation and immunohistochemical analysis

Twelve tissue microarrays were prepared from 200 paraffin NSCLC specimens and dehydrated using graded ethanol after conventional dewaxing, antigen repair, and microwave heating. The endogenous peroxidase blocker was then cultured at room temperature for 10 minutes before being rinsed with PBS. The non-immunized sheep serum was cultured at room temperature for 10 minutes, and the NR2F6 primary antibody (1:100) was cultured overnight at 4°C, then incubated at room temperature for 30 minutes the following day and rinsed with PBS. The biotin-labeled sheep anti-rabbit IgG was cultured at room temperature for 10 minutes and rinsed with PBS. The streptomyces antibiotic protein-peroxidase was cultured at room temperature for 10 minutes and rinsed with PBS. DAB chromogenic reaction; hematoxylin staining; make differentiation with hydrochloric acid ethanol, return to blue with ammonia; dehydrate using graded ethanol, vitrify by dimethylbenzene; and seal with neutral balsam.

Immunohistochemical staining results and staining cell scores: 0 = no positive cells; 1 = positive cells < 10%; 2 = 10–35% positive cells; 3 = 35–75% positive cells; 4 = positive cells > 75%. The grading of staining intensity was based on the following criteria: 0 = no staining; 1 = weak staining (light yellow); 2 = moderate staining (yellowish brown); 3 = strong staining (brown). The staining index (SI) was calculated as the product of the staining intensity score and the proportion of positive cells. Samples with SI ≥ 6 were defined as having high expression, and those with SI < 6 were defined as having low expression.

### Co-immunoprecipitation assay

Cells were lysed for 30 minutes in an immunoprecipitation (IP) lysis buffer containing a protease inhibitor (Roche Diagnostics, Germany). After centrifugation at 13,000 rpm for 15 minutes at 4°C, one tenth of the supernatant was collected as the input group, and half of the remaining supernatant was incubated with anti-flag beads in a rotary mixer at 4°C for 4 hours or overnight; the anti-flag beads were then cleaned with IP lysis buffer three times. The bound proteins were boiled in a 2 × loading buffer, and further analysis was undertaken using Western blotting.

### Statistical analysis

SPSS 24.0 and Graphpad Prism 8 were used for data analysis and statistical chart making. All measurement data were expressed as mean ± standard deviation (mean ± SD) and compared using a *t*-test. A rank sum test was used for ranked data, and the correlation between NR2F6 and clinical correlation analysis of patients by χ^2^ test or Fisher’s exact test. *P* < 0.05 was considered statistically significant.

## Results

### Expression of NR2F6 in lung cancer tissues and cells

**Figure 1:**
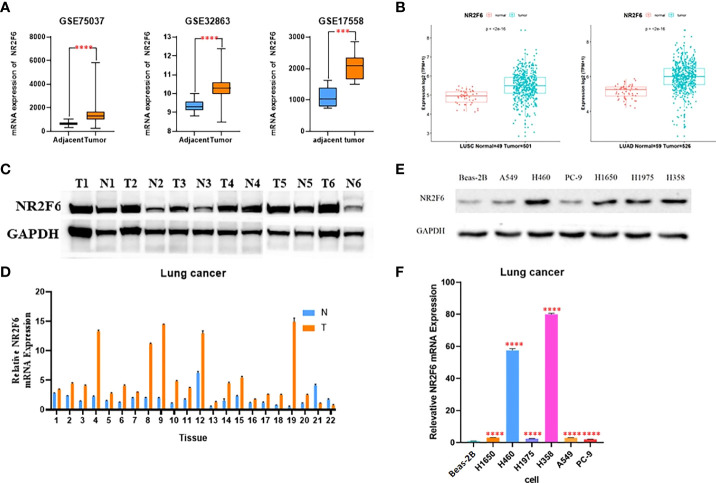
NR2F6 is highly expressed in lung cancer tissues and cells. **(A)** The analysis results of the GSE32863, GSE75037, and GSE17558 data sets showed that the expression of NR2F6 in lung cancer tissues was significantly higher than that in the paired para-carcinoma tissues. **(B)** The Cancer Genome Atlas database showed that the expression level of NR2F6 in lung adenocarcinoma and squamous cell lung carcinoma was significantly higher than that in the paired para-carcinoma tissues, and the difference was statistically significant (*P* < 0.05). **(C)** A Western blot assay was used to detect NR2F6 protein levels in five pairs of lung cancer tissues and the corresponding normal specimens. **(D)** A quantitative polymerase chain reaction (qPCR) assay was used to test NR2F6 transcription levels in 22 pairs of lung cancer tissues and the corresponding normal tissues. **(E)** A Western blot assay was used to detect the expression levels of NR2F6 protein in normal lung epithelial cell line BEAS-2B and six lung cancer cell lines. **(F)** The qPCR test results showed that the expression in lung cancer cells was significantly higher than that in normal lung epithelial cells, and the difference was statistically significant. The above test results are the mean ± standard deviation of three independent repeated experiments. ***P < 0.001, ****P < 0.0001.

As shown in **Figure 1A** of the analysis results of the GSE32863, GSE75037, and GSE17558 data sets, the expression level of NR2F6 in lung cancer tissues was significantly higher than that in para-carcinoma tissues. The TCGA database analysis results in **Figure 1B** show that the expression level of NR2F6 in lung adenocarcinoma and squamous cell lung carcinoma was significantly higher than that in para-carcinoma tissues. In addition, the Western blotting and qPCR results (**Figures 1C, D**) showed that the protein expression and mRNA level of NR2F6 in lung cancer tissues were significantly higher than those in para-carcinoma tissues.

The protein and mRNA levels of NR2F6 in normal lung epithelial cell line Beas-2B and six lung cancer cell lines were further detected. As shown in **Figures 1E, F**, the protein and mRNA levels of NR2F6 in lung cancer cell lines A549, H1650, PC-9, H460, H1975, and H358 were significantly higher than those in Beas-2B. Therefore, adenocarcinoma H358 and large cell carcinoma H460 with the highest expression levels of NR2F6 were selected as the stable lung cancer cell lines for the subsequent construction of the knockdown model of NR2F6.

### The relationship between NR2F6 expression in lung cancer tissues and the clinicopathological features and prognosis of NSCLC

**Figure 2:**
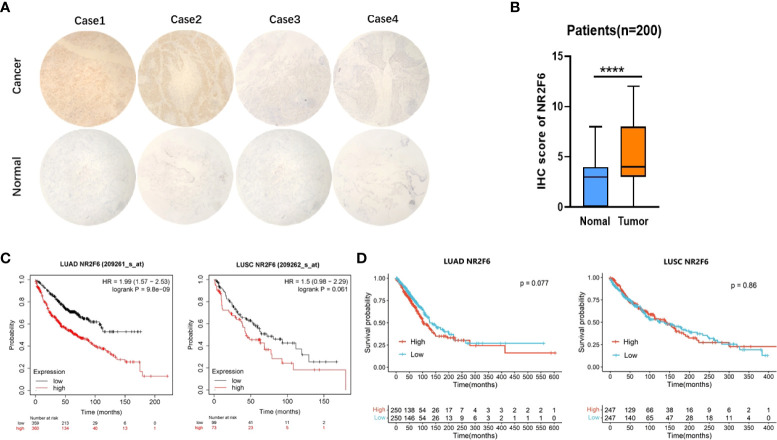
The expression of NR2F6 in lung cancer tissues was significantly higher than that in para-carcinoma tissues and was associated with poor prognosis. **(A)** Immunohistochemical results. The above four images show lung cancer tissues, representing strong positive, positive, weak positive, and negative staining, respectively. The four images below show the corresponding staining results of para-carcinoma tissues. **(B)** The immunohistochemical results showed that NR2F6 protein expression in lung cancer tissues was significantly higher than that in para-carcinoma tissues, and the difference was statistically significant. **(C)** The Kaplan–Meier Plotter was used to analysis the NR2F6 expression and prognosis of LUAD and LUCS. **(D)** TCGA datebase was used to analysis the NR2F6 expression and prognosis of LUAD and ****P < 0.0001.

According to the immunohistochemical analysis results shown in **Figures 2A, B**, the expression level of NR2F6 in lung cancer tissues was significantly higher than that in para-carcinoma tissues (*P* < 0.05). The results in **Table 1** show that the protein level of NR2F6 increased with the progression of tumor invasion depth, clinical stage, and lymph node metastasis, suggesting that a high expression level of NR2F6 is associated with the invasion and metastasis of lung cancer. The Kaplan–Meier Plotter and TCGA datesets were used to analyze the correlation between the expression level of NR2F6 in LUAD and LUSC and the prognosis of patients. The Kaplan–Meier Plotter results (see **Figure 2C**) show that a high expression level of NR2F6 in LUAD is significant associated with poor prognosis (P < 0.05) while TCGA datebase (see **Figure 2D**) show that a high expression level of NR2F6 in LUAD is associated with poor prognosis (P = 0.077). The Kaplan–Meier Plotter results (see **Figure 2C**) show that a high expression level of NR2F6 in LUSC is associated with poor prognosis (P = 0.061) while TCGA datebase (see **Figure 2D**) show that a high expression level of NR2F6 in LUAD is not associated with poor prognosis (P = 0.86)

**Table 1:** Relationship between expression of NR2F6 in lung cancer tissues and clinicopathological features of lung cancer.

Items	NR2F6			p Value (*P < 0.05)
	Total cases (n=200)	Low expression (n=130)	High expression (n=70)
**Age(year)**				
≤60	72	44	28	0.387
>60	128	86	42	
**Gender**				
Male	110	70	40	0.655
Female	90	60	30	
**Histological types**				
Adenocarcinoma	178	68	110	
Squamous cell carcinoma	22	2	20	0.004*
**T stage**				
T1	158	148	10	
T2	32	27	5	0.032*
T3 + T4	12	9	3	
**N stage**				
N0 + N1	168	64	104	0.035*
N2 + N3	32	6	26	
**M stage**				
M0	197	130	67	
M1	3	0	3	/
**Clinical stages**				
I + II	154	62	86	0.004*
III + IV	46	8	38	

### The knockdown of NR2F6 and inhibition of the proliferation of H460 and H358 lung cancer cells

**Figure 3:**
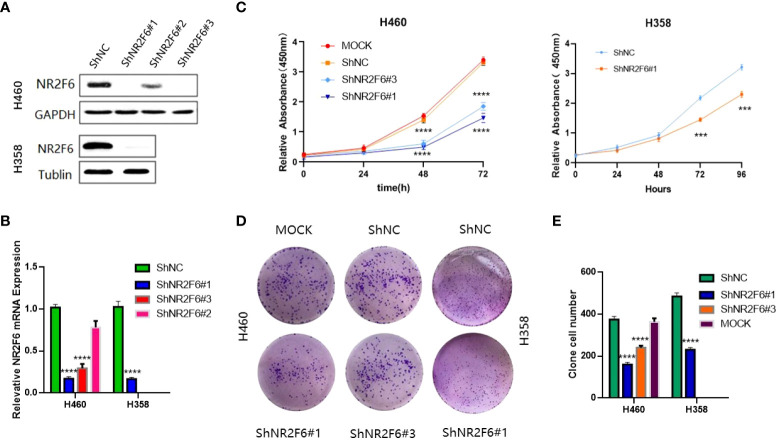
Knockdown of NR2F6 to inhibit the proliferation of H460 and H358 lung cancer cells. **(A)** Western blot test of NR2F6 protein levels in H460 and H358 cells and stale knockdown efficiency. **(B)** Quantitative polymerase chain reaction test of NR2F6 transcriptional level in H460 and H358 cells and stale knockdown efficiency. **(C)** The CCK8 results showed that the knockdown of NR2F6 in H460 and H358 cells significantly inhibits cell proliferation. **(D)** The knockdown of NR2F6 can reduce the clonality of H460 and H358 cells. **(E)** Statistical chart of cell count formed by clone. The above test results are the mean ± standard deviation of three independent repeated experiments. The mock group is the untransfected virus group. shNC is the NR2F6 knockdown control group. shNR2F6#1 and shNR2F6#3 are NR2F6 stable knockdown groups. ***P < 0.001, ****P < 0.0001.

Lung cancer cell line H460 was transfected with lentivirus knockdown by shNR2F6 (a total of three knockdown sequences: shNR2F6#1, shNR2F6#2, and shNR2F6#3). Western blotting (**Figure 3A**) and qPCR tests (**Figure 3B**) showed that the protein expression and mRNA levels of NR2F6 in H460 cells were significantly decreased after transfection with shNR2F6 lentivirus, with shNR2F6#1 knockdown efficiency being the highest, followed by shNR2F6#3. H358 cells were transfected with the shNR2F6#1 sequence, Western blotting and qPCR were used to verify that protein and mRNA levels were both significantly decreased after the knockdown of NR2F6 (**Figures 3A, B**).

In order to verify the effect of NR2F6 on the proliferation of lung cancer cells, a CCK8 kit was used to detect the proliferation of H460 cells in the untreated group (non-transfected mock group), the NR2F6 negative control group (shNC group), and the NR2F6 stable knockdown groups (shNR2F6#1, shNR2F6#3). As shown in **Figure 3C**, the proliferation ability of H460 cells in the shNR2F6#1and shNR2F6#3 groups was significantly weaker than that of the mock group and the shNC group, and the proliferation of shNR2F6#1 and shNR2F6#3 cells was inhibited after 48 hours. The difference was statistically significant (****P < 0.0001). As shown in **Figure 3C**, the proliferation ability of H358 cells in the shNR2F6#1 group was significantly weaker than that of shNC group,which is consistent with the biological function of H460 cells.indicating that NR2F6 knockdown could contribute to the reduction of cell proliferation.

A clone formation assay was also used for the evaluation of cell proliferation, finding that the knockdown of NR2F6 in H460 and H358 cells could effectively inhibit the clone formation of H460 and H358 cells (**Figures 3D, E**; *P* < 0.01).

These above findings further confirm that NR2F6 is closely associated with the occurrence and development of NSCLC.

### The possible interaction between heterogeneous nuclear ribonucleoprotein HNRNPD and NR2F6

**Figure 4:**
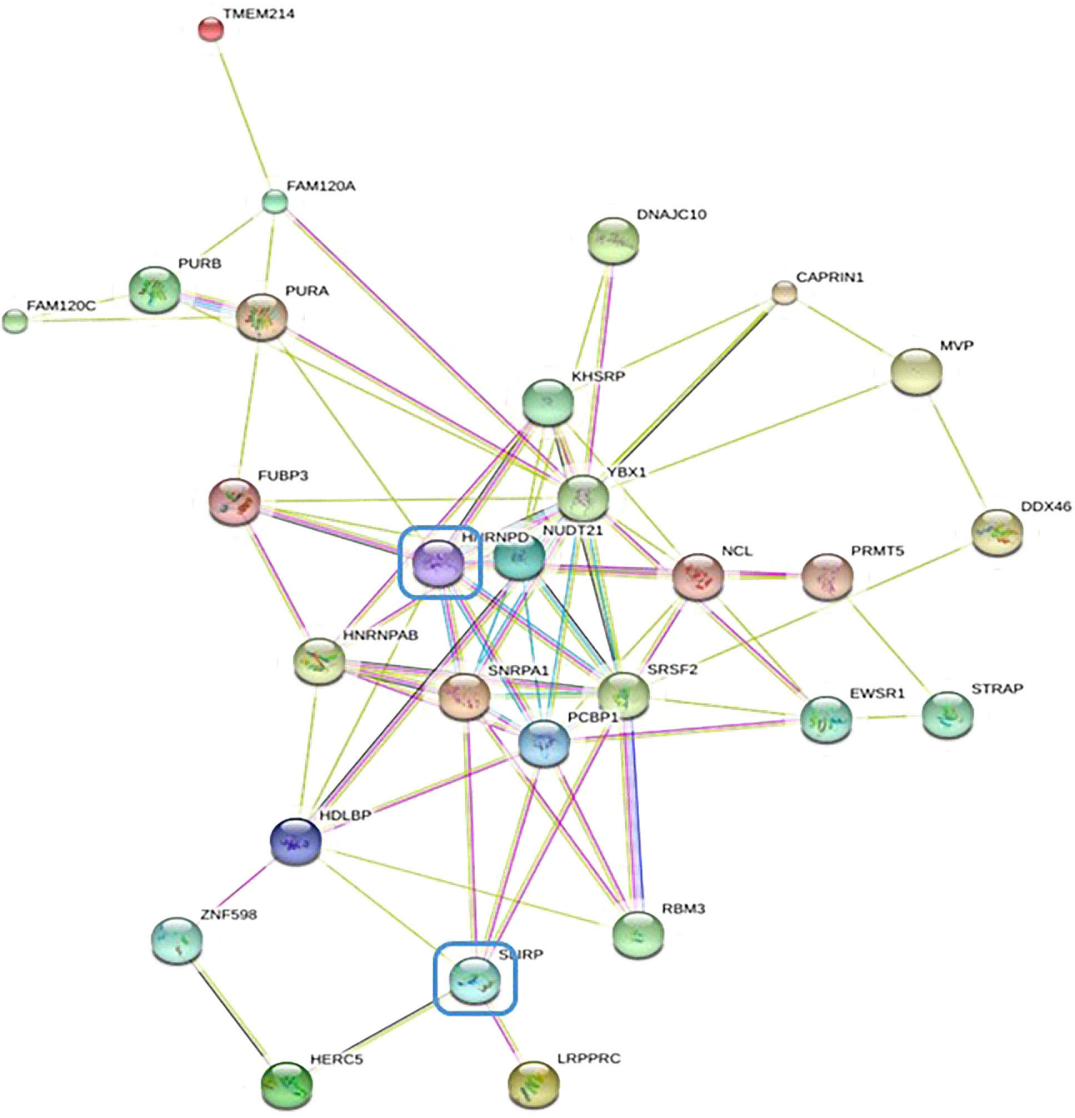
STRING database protein–protein interaction network diagram of differentially expressed genes by mass spectrometry.

In order to further investigate the molecular mechanism of NR2F6 in lung cancer, an immunoprecipitation test was conducted on flag proteins in H460 cells transfected with Flag-NR2F6 lentivirus, after which the protein bound to Flag-NR2F6 was detected using mass spectrometry. The mass spectrometry analysis results (**Figure 4**) revealed that 28 proteins interacted with NR2F6, from which the key protein that could promote the progression of lung cancer—HNRNPD—was screened out.

### The expression of HNRNPD in lung cancer tissues and its correlation with the expression of NR2F6

**Figure 5:**
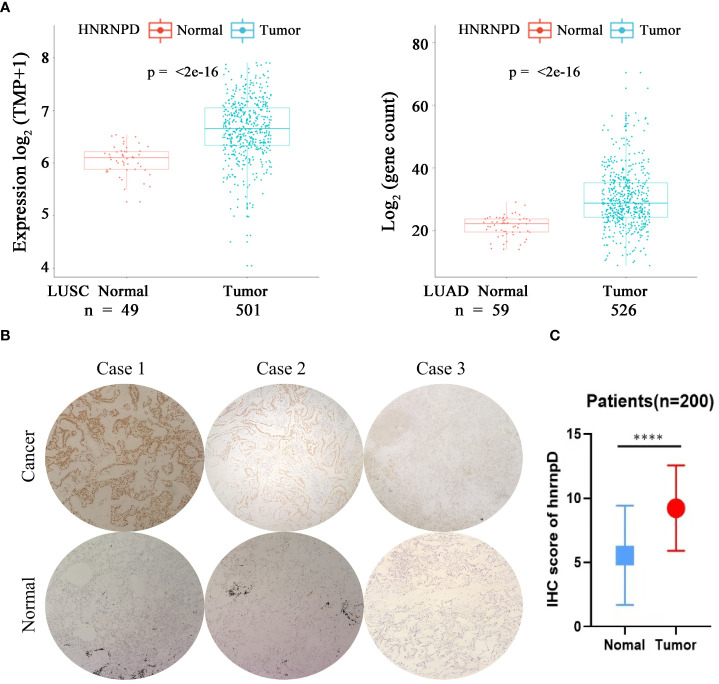
The expression of HNRNPD in lung cancer tissues was significantly higher than that in para-carciooma tussers. **(A)** The Cancer Genome Atlas database showed that the expression of NR2F6 in lung adenocarcinoma and squamous cell lung carcinoma was significantly higher than that in the paired para-carcinoma tissues, and the difference was statistically significant (*P* < 0.05). **(B)** Representative images of immunohistochemical results of HNRNPD in lung cancer tissues. The three images above show lung cancer tissues, representing positive, weak positive, and negative staining. The three images below show the corresponding staining results of para-carcinoma tissues. **(C)** The immunohistochemical results showed that HNRNPD protein expression in lung cancer tissues was significantly higher than that in para-carcinoma tissues, and the difference was statistically significant. *****P < 0.0001.

A high expression of NR2F6 in lung cancer tissues was verified in the TCGA database (**Figure 1B**). In order to study the roles of HNRNPD and NR2F6 and their correlation in lung cancer, the TCGA database and immunohistochemical analysis were further used for verification. The TCGA database results (**Figure 5A**) indicated that the expression level of HNRNPD in lung cancer tissues was higher than that in para-carcinoma tissues. Immunohistochemical staining was performed on tissue chips from continuous sections (200 lung cancer tissues), the immunometric results of which are shown in **Figures 5B, C**. These results also found that the expression level of HNRNPD in lung cancer tissues was significantly higher than that in para-carcinoma tissues, with the difference being statistically significant. The TCGA database and immunohistochemical results showed that HNRNPD was highly expressed in lung cancer tissues and was highly consistent with NR2F6 expression in these tissues.

### The interaction between NR2F6-coded protein and HNRNPD-coded protein

**Figure 6:**
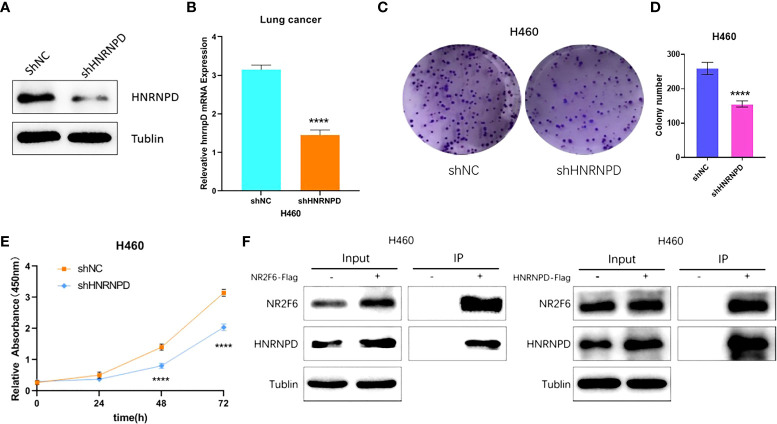
HNRNPD may interact with NR2F6 to jointly regulate lung cancer progression. **(A)** Western blot test of HNRNPD protein levels in H460 cells and stale knockdown efficiency. **(B)** Quantitative polymerase chain reaction test of HNRNPD mRNA levels in H460 cells and knockdown efficiency. **(C, D)** The knockdown of NR2F6 could reduce the clonality of H358 lung cancer cells. **(E)** The CCK8 results showed that the knockdown of NR2F6 significantly inhibited cell proliferation. **(F)** Co-immunoprecipitation verification of NR2F6 and HNRNPD interaction. *****P < 0.0001.

As the results showed that the knockdown of NR2F6 could inhibit the proliferation of lung cancer cells (**Figure 3**), the effect of the knockdown of HNRNPD on the proliferation of lung cancer cells was further investigated. As shown in **Figures 6A, B**, the protein expression and mRNA levels of HNRNPD in H460 cells were both significantly decreased after transfection with shNR2F6 lentivirus. A CCK8 kit was also used to detect the proliferation of H460 cells in the HNRNPD negative control group and the HNRNPD knockdown group. As shown in **Figures 6C, D**, the knockdown of HNRNPD could significantly reduce the clonogenesis ability of H460 lung cancer cells, and the results in **Figure 6E** show that the knockdown of HNRNPD could significantly reduce the proliferation ability of H460 lung cancer cells.

The interaction between NR2F6 and HNRNPD in H460 cells was verified using a CO-IP assay. Flag-negative control and flag-NR2F6 protein with lentivirus stable cell lines were transfected into H460 cells to construct NR2F6 control and NR2F6-flag cells. IP was carried out using anti-flag antibodies. The results on the left of **Figure 6F** show that the anti-flag antibodies co-precipitated the HNRNPD protein in the cells of the NR2F6-flag group but not those in the corresponding NR2F6 control group. The results on the right of **Figure 6F**, however, show that anti-flag antibodies co-precipitated NR2F6 protein in the HNRNPD-flag group but not those in the corresponding HNRNPD control group.

## Discussion

As one of the most common malignancies, and the one with the highest mortality rate (1, 5, 23), the early diagnosis and treatment of lung cancer is vital. It is therefore of great significance for its early diagnosis, treatment, and prognosis to identify an effective therapeutic target. The present study aimed to explore the role and molecular mechanism of NR2F6 in NSCLC in order to provide an experimental foundation for the targeted therapy of NSCLC. The study found that NR2F6 was highly expressed in lung cancer and that the knockdown of NR2F6 could inhibit the proliferation of lung cancer cells. The analysis results of protein CO-IP combined with mass spectrometry revealed the interaction between HNRNPD and NR2F6. The study also found that HNRNPD was highly expressed in lung cancer tissues and that its high expression level was highly consistent with the expression distribution of NR2F6. Furthermore, it was found that the knockdown of HNRNPD can inhibit the proliferation of H460 lung cancer cells, and the interaction between NR2F6 and HNRNPD-coded proteins was confirmed by IP. These findings indicate that NR2F6 may interact with HNRNPD to jointly regulate the progression of lung cancer, providing a new experimental foundation for the study of the molecular targeted therapy of NSCLC.

By analyzing the expression of NR2F6 and the clinicopathological characteristics of lung cancer patients, the present study identified that the expression of NR2F6 was positively correlated with lymph node metastasis, clinical stage, and depth of tumor invasion, suggesting that the high expression level of NR2F6 might promote the invasion and metastasis of lung cancer. This finding is consistent with previous reports on cervical cancer (24), which found that the expression of NR2F6 was significantly correlated with the clinical stage, survival status, tumor recurrence, chemotherapy, and lymph node metastasis of cervical cancer.

The Kaplan–Meier Plotter results and TCGA datebase show that a high expression level of NR2F6 in LUAD is associated with poor prognosis.However, The Kaplan–Meier Plotter results show that a high expression level of NR2F6 in LUSC is associated with poor prognosis while TCGA datebase show that a high expression level of NR2F6 in LUAD is not associated with poor prognosis. The expression of NR2F6 and the prognosis of LUSC need to be further expanded sample research,and our NSCLC patients are being followed up. Previous studies (24) have found that the high expression level of NR2F6 is associated with poor prognosis in various malignant tumors, such as ovarian cancer, early cervical cancer, and head and neck cancer.

The cell experiments in the present study demonstrated that the knockdown of NR2F6 can inhibit the proliferation of lung cancer cells. Jin et al. (25) found that a microRNA can target NR2F6 to inhibit the proliferation, migration, and invasion of lung adenocarcinoma cells and enhance cell autophagy. In a study of colon cancer, Li et al. (18) found that the knockdown of NR2F6 can induce the apoptosis of colon cancer cells by inhibiting X-linked apoptosis protein and that the overexpression of NR2F6 can resist apoptosis induced by the chemotherapy drug etoposide. Ichim et al. (15) found that the stable overexpression of NR2F6 can increase the proliferation of leukemia U937 cells and prevent proliferation, stagnation, and terminal differentiation, while the knockdown of NR2F6 can promote the terminal differentiation of leukemia U937 cells. Li et al. (20) found that the overexpression of NR2F6 can promote the phenotype of ovarian cancer stem cells, while the knockdown of NR2F6 can inhibit it and make ovarian cancer cells sensitive to chemotherapeutics. Wang et al. (26) found that circRH can recruit TIP60 to NR2F6 promoter, activating the expression of NR2F6 and ultimately contributing to the progression of liver cancer, while the knockdown of NR2F6 can inhibit the activation of the notch2 pathway and inhibit the proliferation, invasion, and migration of primary liver cancer cells.

Many of the studies on the molecular mechanism of NR2F6 as a transcription factor focus on protein–protein interaction, but the present study focused on the proteins interacting with NR2F6. Previous studies (27) found that NR2F6 can interact with RUNX1, a key hematopoietic transcription factor, to inhibit its transcriptional activity in mouse myeloblast 32Dcl3. In a yeast two-hybrid screening experiment in 2011, Tan et al. (28) found that NR2F6 can bind to RASD1, and RASD1 can significantly prevent NR2F6 from inhibiting the transcription of the renin gene.

HNRNPD is a member of the heterogeneous nuclear ribonucleoproteins family. Previous studies (29, 30) suggest that it plays a role in promoting tumor progression in a variety of malignant tumors, such as thyroid cancer, oral cancer, and esophageal cancer. Wang et al. (31) reported that HNRNPD can stabilize ZEB1 to induce epithelial-mesenchymal transformation, thereby promoting the development of thyroid cancer; it can also stabilize oncogene c-myc, thereby promoting tumorgenesis (32).

## Conclusion

The present study demonstrated that NR2F6 can promote the proliferation of lung cancer cells at the cellular level and further explored its molecular mechanism, both of which are of important clinical significance for improving the prognosis of lung cancer and identifying new molecular targeted drugs.

## Data availability statement

The raw data supporting the conclusions of this article will be made available by the authors, without undue reservation.

## Ethics statement

The studies involving human participants were reviewed and approved by This study was conducted with approval from the Ethics Committee of the First Affiliated Hospital of Wenzhou Medical University (No : YS2018-106). The patients/participants provided their written informed consent to participate in this study.

## Author contributions

Conception and design of the research: SLY, HQG, LXW, XYH. Acquisition of data: SLY, HQG, HBY. Analysis and interpretation of the data: HBY. Statistical analysis: YC, LC. Obtaining financing: ZFS, LXW. Writing of the manuscript: SLY, HQG. Critical revision of the manuscript for intellectual content: LXW, ZFS, XYH. All authors read and approved the final draft.

## Acknowledgments

This work was financially supported by the Medical Scientific Research Fund of Zhejiang Province (2019322308), and Wenzhou science and technology project (Y20190179).

## Conflict of interest

The authors declare that the research was conducted in the absence of any commercial or financial relationships that could be construed as a potential conflict of interest.

## Publisher’s note

All claims expressed in this article are solely those of the authors and do not necessarily represent those of their affiliated organizations, or those of the publisher, the editors and the reviewers. Any product that may be evaluated in this article, or claim that may be made by its manufacturer, is not guaranteed or endorsed by the publisher.

## Abbreviations

NR2F6, Nuclear receptor subfamily 2 group F member 6; HNRNPD, Heterogeneous nuclear ribonucleoprotein D; qPCR, Real time quantitative polymerase chain reaction; TCGA, The Cancer Genome Atlas; CO-IP, Co-Immunoprecipitation; GEO, Gene Expression Omnibus; WB, Western blot; NSCLC, non small cell lung cancer; SCLC, small cell lung cancer; COUP.TFs, Chicken ovalbumin upstream promoter-transcription factops.
